# Breaking new frontiers: Assessment and re-evaluation of clinical trial design for nutraceuticals

**DOI:** 10.3389/fnut.2022.958753

**Published:** 2022-09-23

**Authors:** Malkanthi Evans, Erin D. Lewis, Joseph M. Antony, David C. Crowley, Najla Guthrie, Jeffrey B. Blumberg

**Affiliations:** ^1^KGK Science Inc., London, ON, Canada; ^2^Friedman School of Nutrition Science and Policy, Tufts University, Boston, MA, United States

**Keywords:** nutraceuticals, methodology, dietary supplement, clinical trials, evidence-based nutrition

## Abstract

Despite sophisticated study designs and measurement tools, we have yet to create an innovative space for diet and dietary supplements in the health care system. The path is challenging due to current hierarchies of scientific evidence and regulatory affairs. The role of the randomized, double-blind, placebo-controlled clinical trial (RCT) as a research approach functions well to characterize the benefits and risks of drugs but lacks the sensitivity to capture the efficacy and safety of nutraceuticals. While some facets of RCTs can be relevant and useful when applied to nutraceuticals, other aspects are limiting and potentially misleading when taken in their entirety. A differentiation between guidelines for evidence-based medicine and the evidence required for nutrition spotlight the need to reconceptualize constituents of the RCT and their applicability with relevance to health promotion. This perspective identifies the limitations of the traditional RCT to capture the complexities of nutraceuticals and proposes the N-of-1 as Level 1 evidence better suited for the proof of efficacy of nutraceuticals.

## Introduction

Nutraceuticals, such as dietary supplements, are defined as products isolated or purified from foods that are generally sold in medicinal forms not usually associated with food and demonstrated to have a physiological benefit or provide protection against chronic disease (1). However, due to their multifunctional nature, study of their mechanisms of action, safety, and efficacy through clinical studies is challenging and new approaches to their investigation are warranted (2).

From a regulatory perspective, nutraceuticals are categorized under a variety of terms, depending on the country's regulatory framework. For example, in Canada there are natural health products (NHPs) and supplemented foods, under which nutraceuticals can be included. The European Food Safety Authority (EFSA), US Food and Drug Administration (FDA), and Health Canada use evidence-based reviews to evaluate the strength of scientific evidence that support marketing claims for foods and dietary supplements, specific to the terms used by each country. This review process assesses causation and is largely based on evidence-based medicine (EBM), a concept where the underlying hypothesis is that the intervention mitigates a condition in a causal manner (3). EBM aims to integrate the best available evidence into the decision-making process. Central to the concept of EBM is the RCT, which permits strong causal inference between an intervention and its outcome. The RCT is central to the regulatory process for both the approval of drugs and health claims of nutraceuticals, making it a critical piece in the evaluation of these products.

This perspective will discuss limitations of the RCT and will show that the traditional RCT drug model is neither sensitive nor relevant to nutraceuticals because (i) true placebos are not possible, (ii) effect sizes in ‘healthy' volunteers are most often modest, (iii) statistical analyses need to be refocused, (iv) endpoints must be global or multifunctional, (v) proof of efficacy should advance to that of probable harm due to lack of the intervention, and (vi) of the requirement for ‘healthy' participant enrollment. Therefore, we propose N-of-1 studies as Level 1 evidence better suited for nutraceuticals.

## The RCT drug model is neither sensitive nor relevant to nutraceuticals

Currently over 400,000 RCTs, are being conducted throughout the US and in 220 other countries (4). Case series studies, case reports, and big data continue to inform routine care of patients (5–8). For example, adoption of some surgical techniques were not based on RCTs but compelling visual evidence (9, 10). It was estimated that 49% of drugs approved by FDA during 2005–2012 were based on “surrogate endpoints” rather than clinical outcomes (11). This suggests that biochemical changes that may or may not lead to clinical improvements were also found to be sufficient for approval of drugs despite the lack of clinical evidence (11).

Areas of research such as in psychotherapy where individualized interventions cannot be generalized or applied via the RCT have been accepted in treatment modalities (12, 13). Previously, RCTs failed to inform accurately, examples being tolbutamide (anti-diabetic drug), which led to secondary failure of response in patients (14) and the ALLHAT trial on thiazide diuretics (15, 16). The “shaky conclusions” of ALLHAT studies that were cloaked in EBM reported chlorthalidone as superior to the existing “gold” standard diuretics (17). Results from RCTs incorporated into care were later found to be inaccurate or insufficient for reliance (18). However, systematic reviews and meta-analyses are considered the new gold standard (19). Bothwell et al. stated “even though RCTs were developed to produce generalizable and universal knowledge, they have remained entangled in local social, economic and political conditions” (20). Due to their exorbitant cost, publishing positive results became a focus, resulting in an imbalance in medical knowledge, leading to publication-biased evidence (21–24).

Easily obtained victories for pharmaceuticals have been based on crowd-based medicine efforts such as vaccinations and other population-based interventions. Indeed, though RCTs were originally designed to decrease bias in research, they have become a point of “conflicting interest” (25, 26).

Sackett et al. stated: “individual patient care is not restricted to randomized trials and meta-analyses. It involves tracking down the best external evidence with which to answer our clinical questions” (27). However, EBM appears to have been extended into evidence-based nutrition (EBN), making thoughtful and reasonable assessments of nutraceuticals challenging.

The application of RCTs to disciplines not related to testing drugs, such as foods, beverages, and dietary supplements, is fraught with challenges, the most prominent of which are the inherent differences between drugs and nutrients. Drugs are directed toward treatment of disease. They have isolated functions and are designed to target single organs or tissues and are not homeostatically controlled by the body. Unlike most drugs, nutrients work in complex networks, target all cells and tissues, and have multifaceted effects and outcomes. Therefore, RCTs for nutrients must be designed to capture this multifunctionality. Since many nutrients are homeostatically regulated, the body's baseline status affects the response to a nutraceutical intervention. For those not homeostatically regulated, excessive amounts are either not bioavailable, or are metabolized relatively quickly and/or eliminated, thus leading to their generally high level of safety.

### True placebos are not possible in nutrition studies

Large effect sizes can be expected in drug studies, intended to show superiority or comparability, as there is a no-intake control group or a true placebo. There is a distinction in the reduction of the symptoms of disease, which is present in the enrolled population and the absence of the intervention does not cause disease (23, 28).

In contrast, true placebos are not possible with nutraceutical studies and the interventions involve the intake of nutrients, the absence of which may cause disease. In the absence of a true placebo group, and the requirement of ‘healthy' participants, nutrients have a smaller effect size and a longer response time to detect health outcomes. Essential nutrients are necessary for health and thus the underlying hypothesis that low or inadequate intake of nutrients causes or contributes to disease (29). Heaney stated, “with nutrients, the question is always not ‘whether' but ‘how much'?” (29). As such, EBN is a complex and intricate puzzle. Only one function of a nutrient (the first to appear and lead to disease or death) is used to define the disease (i.e., deficiency syndromes like beriberi and scurvy). But our understanding of all the other functions (beyond deficiency) is where we find the challenge of demonstrating the many benefits of a single nutrient when the proof of efficacy is limited to EBM (29).

### Effect sizes are modest: Contending the requirement for ‘healthy' participants

The regulatory requirements of enrollment of populations “defensible as healthy” in studies intended for substantiation of structure/function claims limit the purpose of nutraceutical investigations. This accentuates the small effect size, decreasing the gap between the placebo/control and intervention (30). In the absence of an acceptable definition for ‘healthy,' a regulation that uses such a guideline to reject a study for claims is questionable. Changing medical treatment algorithms (31) that continuously redefine healthy populations while moving the dichotomy in the definition of health vs. disease and advocating earlier pharmacological treatment narrows the population that may be called ‘healthy.' Not only are these restrictions impractical for participant recruitment, but they are also not conducive for moving markers of respective outcomes and highlights flaws in logical application.

Persistent advocacy and requirements result in findings of clinical trials being limited to narrow and unrepresentative populations. This is a disservice to consumers seeking alternative solutions to improve their health. More recently, the concept of ‘healthy' has been upended by investigations showing that those previously considered ‘healthy' may in fact be metabolically unhealthy (32). A paradigm change is needed in defining the term ‘healthy' when applied to the nutraceutical industry.

### Statistical analyses should be refocused: Statistical vs. clinical significance

The tendency of scientists and researchers to rely and focus on *p* values as the “gold standard of statistical validity,” above and beyond other frameworks for data analysis, which include statistical power, false positives and negatives have been challenged (33). The *p* value does not address the question if the probability of the study hypothesis is correct in the first place, and furthermore does not account for the actual size of the effect. Nuzzo argued that “researchers need to realize the limits of conventional statistics” and “bring into their analysis elements of scientific judgment about the plausibility of a hypothesis and study limitation” (33).

Recent statements by the American Statistical Association (ASA), EFSA, and New England Journal of Medicine (New Engl J Med) point away from such requirements that are founded on misinformation and urge movement toward providing clinical significance. “The *p* value was never intended to be a substitute for scientific reasoning,” said Wasserstein, stating “Well-reasoned statistical arguments contain much more than the value of a single number and whether that number exceeds an arbitrary threshold” and is a directional move away from and toward a “post *p* < 0.05 era” (34).

The EFSA Scientific Committee suggested that statistical significance should not be the primary objective in analysis and focus should be directed on confidence intervals and biological relevance (35). New Engl J Med followed with a statement regarding publications and *p* values stating that nutrient studies should be directed to solve a health indication, not for statistical convenience (36).

### Endpoints should be global and multifunctional

The multifunctional nature of nutraceuticals and their multi-targeted outcomes are not readily determined within the scope of the current RCT model. These multifunctional effects could be considered a complex intervention (37) and are not often captured in the setting of a single primary endpoint, resulting in the intervention being found ineffective and leading to higher proportions of false negatives. The Medical Research Council of the United Kingdom stated that complex interventions require a different approach. While a single primary outcome is usually the most straightforward for statistical analysis, it may not provide adequate assessment of the success of an intervention that has effects across a range of indications (37).

A global outcome that assesses and scores the effects of an investigational product across systems corresponds more closely to the multifunctional outcome proposition of nutrients in the human body. When designed to reflect health rather than disease, a global outcome captures systemic effects (37). Previously, such a concept has been proposed as a composite of several outcomes providing a higher statistical power to the study to detect small changes across multiple organ systems (38).

Global indices of health are appropriate in the nutritional sciences realm since functional foods, nutraceuticals, and other NHPs do not fit into the pharmaceutical model. A global index is different from a composite of several outcomes. A composite index cannot identify the variable that contributes to the majority of the effects, and when combined as a primary outcome can also mask a real benefit of a single constituent endpoint by being diluted from multiple null effects within the cluster (39, 40). A global index weights each outcome allowing for a better and more informed assessment of the outcome(s) contributing to the results. Health-related quality of life questionnaires such as the 36-item Short Form Survey (SF-36) or Profile of Mood States (POMS), and objective biomarkers might be examined as an approach for creating global health indices that better capture the efficacy of the intervention. This has been previously reported where Antony et al. suggest combining outcomes of cognition with physiological biomarkers of immunity and metabolism to arrive at a global index for cognitive health (41).

Furthermore, the index should evaluate and quantify positive movement of a negative health marker. Such concepts have previously been evaluated in quantifying disease activity in patients with rheumatoid arthritis and includes patient history, laboratory tests, physical examination, and imaging studies (42). Multiple common indices, were used to assess adiposity, such as body mass index (BMI), bioelectric impedance analysis (BIA), and anthropometric measures like skinfold thickness and waist: hip ratio as well as computed tomography imaging (42). Thus, a global index as the primary outcome comprising multiple single endpoints could improve statistical precision, increase efficiency, reduce trial size and cost, and may provide trial results earlier (43).

### Advancing from proof of efficacy to proof of probable harm

Heaney questioned “whether we need as much proof of efficacy for a nutrition policy decision as we do for approval of powerful, expensive and potentially dangerous pharmaceutical agents” (29).

Reductions in intake of a nutrient may lead to an increase in the risk of disease or may result in disease. It is unethical to lower nutrient levels to study efficacy endpoints. Heaney et al. proposed a shift from examining proof of efficacy to that of probable harm, a calculus of benefit compared to harm, to be evaluated on a nutrient-by-nutrient basis (29). Investigating pre-diseased populations where the placebo or control group presents either low or harmful levels of surrogate biomarkers may be beneficial in the application to nutraceuticals. Using the calculus of benefit vs. harm, the proof of harm is established in a pre-diseased population since they will eventually progress to a diseased state in the absence of an intervention. If the safety of nutraceutical interventions can be demonstrated through high-quality and comprehensive studies, then the calculus of benefit vs. harm of the intervention shifts toward benefit.

A recent study examined this concept in an RCT model and found that following the progression of the placebo group from baseline to the end of the study was valuable. Left untreated, an at-risk population progressed to disease, which was observed in the placebo group (44) confirming Heaney's concept (29). On further investigation and application of the Framingham risk score, it was found that left untreated, those with cardiovascular risk factors may progress to a more hypertensive and hypercholesterolemic state (44).

A small effect size and study populations, as well, the flawed expectations that nutraceuticals should behave like drugs often result in RCTs generating null results. If one were to stop at this point, most RCTs would suggest that the ingredient and or the formulation was not efficacious.

Therefore, application of EBM principles to EBN solely for meeting the standards of acceptable evidence for policy makers is contentious. Applying the principles of EBM to EBN in its entirety is troublesome and unsuitable for the assessment of nutrients, dietary supplements, and foods. A paradigm shift is required in trial design for evaluating nutrient principles and examining RCT designs that are better suited for nutraceuticals. Blanket application of the RCT concept to the nutraceutical model is not sensitive to establish health promotion and optimization and, in many instances, has led to an exercise in futility.

Thus, it would be reasonable to expect that the evidence required to prove efficacy for drugs and nutrients would need to be different. Both drugs and nutrients have different attributes in contributing to the welfare of an individual. Importantly, drugs are therapeutic in nature, whereas nutrients may not only reduce the risk for disease but may help promote optimal health and performance.

### N-of-1 studies: The RCT at level 1 evidence stage

There are various considerations for designing high-quality studies for nutrition and nutraceutical research. Considerations have been previously presented by Lichtenstein et al. and the Agriculture and Agri-Food Canada “Best Practices for Food-Based Clinical Trials” (46, 47). Following rigorous design considerations, there have been large-scale, long-term, double-blind, placebo-controlled trials of dietary supplements including but not limited to the Age-Related Eye Disease Study (AREDS) and AREDS 2 (48), Alpha-Tocopherol, Beta-Carotene Cancer Prevention (ATBC) Study (49), Carotene and Retinol Efficacy Trial (CARET) (50), Cocoa Supplement and Multivitamin Outcomes Study (COSMOS) (51), Selenium and Vitamin E Cancer Prevention Trial (SELECT) (52), Supplementation en Vitamines et Mineraux Antioxydants (SU.VI.MAX) (53), VITamin D and OmegA-3 TriaL (VITAL) (54), Women's Antioxidant and Folic Acid Cardiovascular Study (WAFACS), Physicians' Health Study-I and II, Women's Health Initiative (WHI), and Women's Health Study (WHS). However, these large-scale trials are extraordinarily expensive, not always feasible, and mostly unnecessary in the context of evaluating the health benefits of nutraceuticals.

Previous research in nutraceuticals validates the importance of measuring responsiveness to treatment at the individual level (24). About 25% of participants responded positively to an improvement in blood pressure and approximately 33% to insulin sensitivity (24). An estimated 66-75% of participants were non-responders to the intervention, which indicated that the intervention worked in a particular subset of participants, a group that could not be identified prior to the study. Examination of group variability allows researchers to make informed decisions of the inter-individual differences of clinical relevance (55). Population-wide recommendations, currently in place for reduction in dietary sodium, were based on the benefits in hypertensive subjects and implementation of folic acid fortification programs were based on neural tube defect prevention (56). The evidence demonstrates a precedence exists for nutrition study results in a responder population that can be generalized to the greater population when positioned correctly. Studies on probiotic interventions for antibiotic-induced diarrhea have indicated similar results (57).

The history of N-of-1 show this type of study was developed due to the need for determining “optimal therapy” for a patient and proved to be “spectacularly helpful” in patient treatment (58). Since its publication, clinicians have reported on thousands of N-of-1 trials. These studies improved patient outcomes and drug development, expanded to encompass many indications (59), and have reported their value for conditions where conventional RCTs are available (60). N-of-1 trials are defined in part by randomizing multiple crossover trials in a single participant (61–64). This N-of-1 study design has been utilized or proposed in nutrition and nutraceutical study designs (65–69). For example, the Westlake N-of-1 Trials for Macronutrient Intake (WE-MACNUTR) examined the use of personalized dietary recommendations on glucose metabolism and gut microbiota in the prevention of chronic disease (66, 68). Further, Soldevila-Domenech et al. reviewed the use of nutraceutical interventions, including phenolic compounds, omega-3 polyunsaturated fatty acids, vitamin D, vitamin C and other micronutrients, aimed at improving cognitive function in older adults with the potential application to N-of-1 designs specifically for dementia prevention (69).

**Figure 1:**
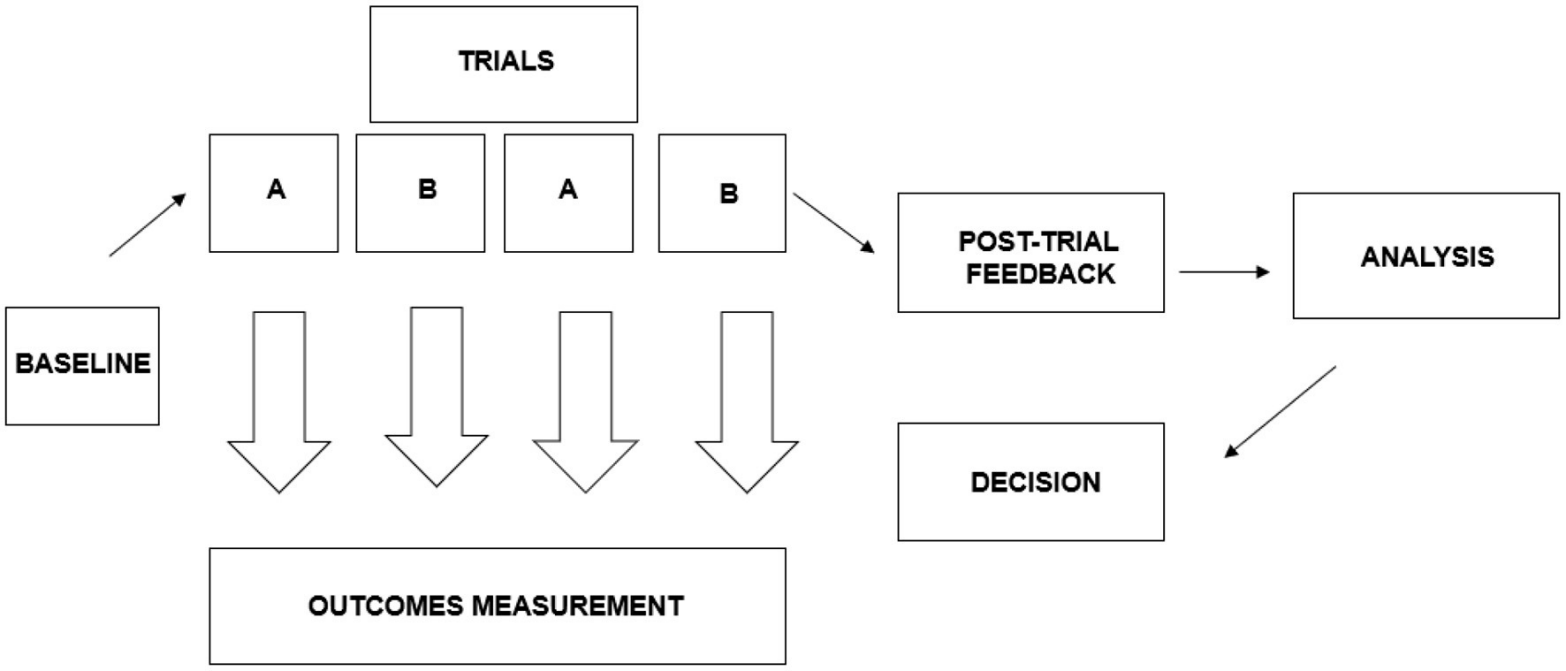
The N-of-1 trial design used in comparing responses to two interventions. Adapted from Zucker et al. (45).

Recently, N-of-1 designs, as part of the umbrella of single-case experimental designs (SCDs), has been discussed in detail by a Nutrition Research Task Force of the American Society for Nutrition (70). They state that “to determine whether, and to what extent, people respond differently to interventions, different designs needs to be used” (70). A general example of an N-of-1 trial design used for comparing responses to two interventions is shown in Figure 1. The intention of this design is to utilize multiple N-of-1 studies in informing decision making.

When compared to the traditional RCT, N-of-1 provides greater strength and more reliable data in clinical decision-making (60). Further, this design may provide specificity and inherently address the heterogeneity of treatment effects when compared to RCTs (70). Models have been expanded to accommodate crossover features including carry-over effects (71) and maintains randomization, blinding, and formal outcome assessment. The Oxford Centre for Evidence-Based Medicine has classified N-of-1 trials as Level 1 evidence, the same level of evidence for systematic reviews of RCTs (72). N-of-1 studies are a unique method identifying optimal intervention approaches for individual cases. Accumulation of multiple N-of-1 studies utilized in meta-analysis can be generalized across investigational populations (60, 70).

The N-of-1 trial allows for rapidly identifying responders vs. non-responders, requires enquiry into clinical significance, and provides a venue for the use of a global index outcome that is more generalizable to the nutraceutical industry (73). Furthermore, an N-of-1 at a Level 1 evidence stage allows for the measurement of probable harm in the absence of an intervention, provides clinical relevance, allows for the accuracy of detection of efficacy, and can function within the guidelines of the traditional RCT. Such a model would be of value in providing reliable and reasonable information regarding the intervention.

## Conclusion

The conventional RCT model does not withstand the scrutiny required to make it useable as a source of evidence for the proof of efficacy of nutraceuticals. The N-of-1 RCT design obviates the limitations and lack of sensitivity of the traditional RCT used to test drugs in the therapeutic milieu. The N-of-1 design provides a more relevant control group, addresses concerns about effect size, can capture global or multifunctional outcomes, allows for advancing from proof of efficacy to that of probable harm in the absence of an intervention, and measures clinical significance. N-of-1 studies provide faster information for identifying optimal individualized interventions, and facilitating translational research by capturing principles that can be generalized to population subgroups (70). Accumulation of multiple N-of-1 studies utilized in meta-analysis can be generalized across investigational populations (70). This Perspective provides an argument for the application of the N-of-1 RCT design, an established Level 1 evidence stage, for proof from interventional studies of nutraceuticals.

## Data availability statement

The original contributions presented in the study are included in the article, further inquiries can be directed to the corresponding author.

## Author contributions

ME and JB contributed to conception and design of the perspective. ME, EL, JA, and JB wrote the first draft of the manuscript. All authors contributed to manuscript revision, read, and approved the submitted version.

## Conflict of interest

Authors ME, EL, DC, JA, and NG are/were employed by KGK Science Inc. The remaining author declares that the research was conducted in the absence of any commercial or financial relationships that could be construed as a potential conflict of interest.

## Publisher's note

All claims expressed in this article are solely those of the authors and do not necessarily represent those of their affiliated organizations, or those of the publisher, the editors and the reviewers. Any product that may be evaluated in this article, or claim that may be made by its manufacturer, is not guaranteed or endorsed by the publisher.
